# Forest loss increases foliar insect and pathogen damage on poplar trees in natural riparian forests

**DOI:** 10.3389/fpls.2025.1508665

**Published:** 2025-06-16

**Authors:** Binli Wang, Ling Li

**Affiliations:** Department of Biological and Food Engineering, Lyuliang University, Lvliang, China

**Keywords:** forest loss, insect herbivory, morphological leaf traits, pathogen infection, tree diversity

## Abstract

Landscape-scale forest loss threats biodiversity and ecosystem functioning, but its effect on insect herbivory and pathogen infection on trees is not well understood. Little is known about how forest loss alters the effects of biotic and abiotic factors on foliar damages. Here, we assessed the relative importance of forest loss, microclimates, tree community attributes, leaf traits (e.g., specific leaf area, SLA), and arthropod abundance on insect and pathogen damage on laurel poplars in natural riparian forests in Xinjiang, China. We found that forest loss increased foliar insect herbivory directly through reduction of food availability and indirectly through reduction in tree diversity and host resistance (greater SLA). In comparison, forest loss only indirectly increased pathogen infection through lower tree diversity and associated higher SLA. Early season insect herbivory promoted later season pathogen infection. Microclimates were not associated with insect and pathogen damage, nor was arthropod abundance with insect herbivory. Our results suggest that forest loss reduced tree diversity which, in turn, changed host leaf traits and associational resistance and undermined bottom-up controls on insect herbivory and pathogen infection. Comparatively, top-down control of herbivory through predation was not significant. The positive relationship between tree diversity and host resistance (leaf traits, e.g., SLA) may be critical for maintaining forest health and ecosystem functioning in plantations and fragmented natural forests when insect pathogen damage at normal, non-outbreak conditions.

## 1 Introduction

Human activities are dramatically changing forests, the largest land biome on earth (Grantham et al., 2020). In forest management, natural forests of high species and structural diversity are often replaced with plantations of low diversity (Brockerhoff et al., 2008). Anthropogenic activities also break large and contiguous forest patches into smaller fragmented patches eventually total loss of forests (Peres et al., 2006; Curtis et al., 2018). Both forest simplification and fragmentation affect forest composition, structure, and ecosystem processes (Echeverría et al., 2007; Haddad et al., 2015). While plantation forests can recover species and structural diversity over time (Hartley, 2002; Bremer and Farley, 2010), the forest fragmentation by agricultural expansion and land-use intensification often lead to permanent changes in forest composition and structure including insects and pathogens (Kehoe et al., 2017; Guégan et al., 2023; Morante-Filho et al., 2024), the core components of forest ecosystems (Boyd et al., 2013).

Fragmented forest patches differ from large and continuous forest patches in composition, structure, and microclimates (Echeverría et al., 2007; Magnago et al., 2015), which would affect growth, health, and regeneration of host trees and thus insect herbivores and pathogens through bottom-up control (Ruiz-Guerra et al., 2010; Altamirano et al., 2016; García-Guzmán et al., 2016; Elderd, 2019). For example, smaller patches have greater edge effects (Didham et al., 2012; Hending et al., 2023) favoring pioneer species and discouraging shade-tolerant species (Laurance et al., 2006; Giriraj et al., 2010; Arroyo-Rodríguez et al., 2016). Pioneer plants are generally fast-growing and their short-lived leaves possess limited defenses against folivorous insects and foliar fungal pathogens (Coley and Barone, 1996; Toome et al., 2010; Schuldt et al., 2017). The altered temperature, moisture, and light regimes can change plant palatability and nutritional quality (Clissold and Simpson, 2015; Stiegel et al., 2017).

Forest fragmentation can also change predation, parasitization, and growth environments and affect herbivorous insects through top-down controls (Roland and Taylor, 1997; Peter et al., 2015). Predator and parasitoid populations generally decline in fragmented forests as sensitivity to habitat patch size increases with trophic position (Rand and Tscharntke, 2007; Fenoglio et al., 2012) and degree of habitat specialization (Lami et al., 2021). The loss of natural enemies in fragmented forests may release prey populations from top-down control (Schüepp et al., 2014; Maguire et al., 2015). However, the extent to which insect herbivores are released from predation may depend on natural enemy’s traits such as dispersal and competitive ability, and diet breadth (Rand and Tscharntke, 2007; Cagnolo et al., 2009; Bellone et al., 2020). In particular, specialist predators and parasitoids that are highly dependent on ecologically specialized prey would be most susceptible to local extinction in fragmented landscapes when their prey is either reduced in abundance or entirely absent (Ryall and Fahrig, 2006; Bitters et al., 2022). Therefore, communities in fragmented forests are likely to be deficient in interactions between ecologically specialized insect herbivores and their natural enemies (Valladares et al., 2012).

Both bottom-up and top-down controls are species-dependent and can be positive, negative, or neutral. For example, insect herbivory can increase (Peter et al., 2015; Castagneyrol et al., 2019), decrease (Ruiz-Guerra et al., 2010; Cavaletto et al., 2019), or do not change (Rossetti et al., 2017; Souza et al., 2013) in responses to change in host trees, predators, and microclimates within fragmented forest patches (Didham et al., 2012; Martinson and Fagan, 2014; Altamirano et al., 2016). Insect herbivory and pathogen infection also interact each other (Tack et al., 2012; Gossner et al., 2021), resulting in complex multitrophic interactions and responses to forest fragmentation (Meentemeyer et al., 2008; Rossetti et al., 2014; Guégan et al., 2023). Therefore, both abiotic and biotic factors need to be assessed to understand bottom-up and top-down influences of forest fragmentation (Vidal and Murphy, 2018), which is often difficult due to reciprocal interactions between insects and pathogens (Franco et al., 2017). To date, most empirical evidence on forest fragmentation effects stems from case studies of outbreaks of particular pest species and from highly controlled experiments (Cavaletto et al., 2019; Bitters et al., 2022). Despite the strengths of evidence, the results of these studies do not reflect the diversity and complexity of drivers that affect insect and pathogen damage under natural, non-outbreak conditions.

Laurel poplar (*Populus laurifolia* Ledeb.) is naturally distributed in Kazakhstan, the Altai, Mongolia, and Xinjiang in China (Proshkin and Klimov, 2017; Wei et al., 2025). In Xinjiang, laurel poplar prefers disturbed riparian areas and is the dominant deciduous tree species in mixed forests along the Irtysh River, providing high symbolic, ecological, and economic importance (Wang et al., 2019). Laurel poplar hosts diverse folivorous insects and often has *Marssonina* leaf spot disease (MLSD) (Wang et al., 2021) from a specialist fungal pathogen *Drepanopeziza populi* (Wang et al., 2022). The insect herbivory and MLSD damage on laurel poplar are generally separated in phenology (Wang et al., 2021), with most folivorous insects occurring in late spring (May-June) and MLSD infection peaking at high temperatures and precipitation in late summer (August-September) (Wang et al., 2022). The intensive land use in Xinjiang (Song et al., 2024) and the host of diverse folivorous insects and leaf diseases make laurel poplar a suitable model species for studying on the influence of forest fragmentation on insect and pathogen damage and their interactions. Specifically, we want to investigate: (1) the effects of forest fragmentation on foliar insect herbivory and pathogen infection, (2) the mediating roles of tree diversity, microclimate, leaf trait, and abundance of arthropod herbivores and arthropod predators, (3) bottom-up and top-down controls over insect herbivory and pathogen damage in fragmented forest patches. Our ultimate goal is to improve understanding of bottom-up and top-down mechanisms that may influence effects of forest fragmentation on insect and pathogen damage.

## 2 Materials and methods

### 2.1 Study area and plot selection

**Figure 1:**
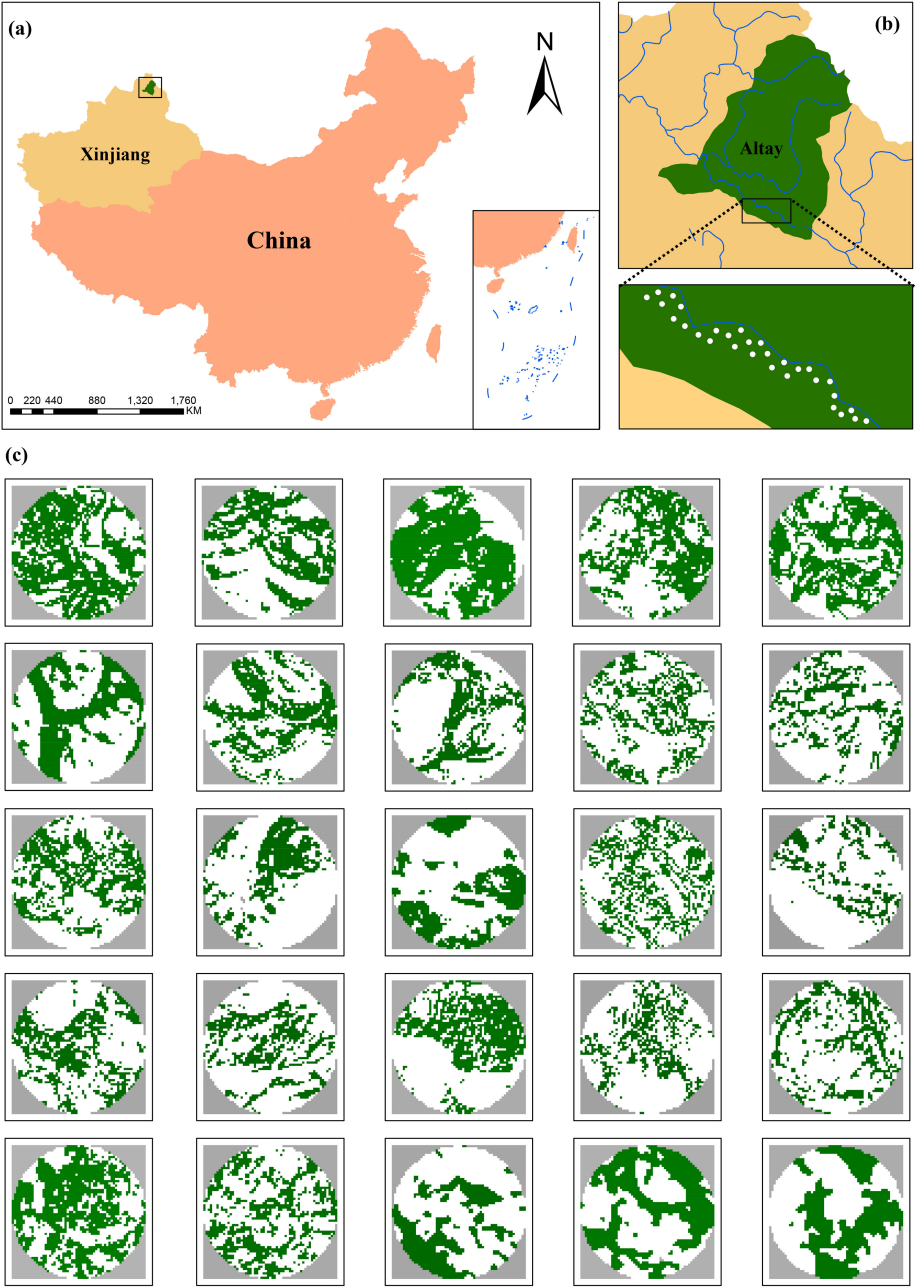
**(a)** Location of study area in northern Xinjiang, North-western China. **(b)** Spatial distribution of 30 sampling plots (white points) in natural forests along the Irtysh River. **(c)** The mosaics of land-cover types within a 300-m radius buffer surrounding the center of each sampling plot, including poplar woodland (green areas) and non-woodland (white areas).

This study was carried out in natural forests along the Irtysh River in northern Xinjiang, North-western China (**Figure 1a**). Due to high latitude, this region has a unique temperate continental climate characterized by a dry season with lower humidity and precipitation and higher temperatures from May to September, without a defined wet season (Wang et al., 2022). The anthropogenic changes in the last five decades has transformed natural forests into a mosaic of different land-cover types, including poplar woodlands, grasslands, agricultural areas, wastelands, and scattered human settlements (Hrkal et al., 2006). In particular, disturbances such as hydraulic engineering, land-use alteration, and grazing have significantly increased vulnerability, which leads to 15~20% total loss of forests, a continuous reduction in woodland coverage, and a decline in the value of ecosystem services, and endangerment of numerous plant species (Song et al., 2024). The laurel poplar is predominating in the woodlands of the study area, accompanied by *Populus alba* L., *Populus nigra* L., and *Populus* × *jrtyschensis* C. Y. Yang (Wang et al., 2019).

To facilitate site selection, all poplar woodland in the study area were mapped using geo-rectified MSI imagery acquired by the European Space Agency’s Sentinel-2A satellite on July 27, 2023. The MSI imagery consists of four spectral bands (red, green, blue, and near infrared), and is appropriate for land-cover classification of diverse vegetation types (e.g., poplar woodland, farmland, and grassland) using ENVI 5.3 (Exelis VIS, USA). To confirm the suitability of potential study sites, we visually verified in the field, especially the presence of laurel poplars. In total, we established 30 sampling plots (30 × 30 m) in poplar woodlands within the study area using ArcGIS 10.5 (ESRI, USA) (**Figure 1b**). The sampling plots varied in levels of forest cover and located at least 600 m apart to avoid spatial correlation between insects and *D. populi* (Wang et al., 2019, 2022). Moreover, the geographical extent of study area and the distance “600 m” were designed to quantify landscape-scale forest loss.

Laurel poplar in the study area is naturally distributed and grows primarily on the floodplain of the river alluvium where sandy soils are characterized by lower clay content, moisture retention capacity, organic matter, and nutrient retention, but higher pH, CaCO_3_, and infiltration. These physical and chemical properties of the soils are thus relatively consistent. As artificial floods caused by “635” hydraulic engineering only last for about a week annually, the sandy soils in the floodplain have a relatively low soil moisture (about 10%~15%). Additionally, poplar woodlands were primarily located in low-elevation (about 500 m) plain valley areas with a relatively flat terrain. Therefore, changes in elevation, soil properties, and soil moisture were not considered as potential sources of significant variations in plants, insects, and diseases.

### 2.2 Forest loss

We quantified forest cover in a circular area of 300 m radius surrounding each sampling plot using a sample site-landscape approach (Fahrig, 2013). We classified land-cover types into non-woodlands and woodlands by processing Sentinel-2A imagery (**Figure 1c**) and calculated forest loss as the percent of non-woodland area within a 300-m radius from each plot. The “300 m radius” area was selected, as this spatial scale can capture the influences of landscape variables on insect and pathogen damage (Wang et al., 2021) and is relevant for abundance of insect herbivores and forest specialist predators (Wang et al., 2019). This landscape size is large enough to reflect variations in the explanatory variable (**Figure 1c**) and to reduce spatial overlaps between adjacent plots required for accurate ecological inferences about biological responses (Eigenbrod et al., 2011).

### 2.3 Tree community survey

Host density and tree diversity within each sampling plot were calculated to assess the effects of tree community attributes on insect and pathogen damage. Both variables are strongly associated with forest cover (Meentemeyer et al., 2008; Rocha-Santos et al., 2016) and the levels of leaf damage (Hantsch et al., 2013; Castagneyrol et al., 2017). Within each plot, we recorded the identity and number of all trees with a diameter at breast height (DBH) ≥ 5 cm. Host density was estimated as the number of host trees per hectare based on the number of laurel poplars in the plot area (900 m^2^). Tree diversity was estimated by calculating the Shannon’s diversity index of tree species in each plot using the R package *vegan* (Oksanen et al., 2022).

### 2.4 Arthropod surveys

Arthropods on laurel poplar trees and understory vegetation were surveyed in late spring when arthropods generally peak in abundance. Within each plot, five host poplars were randomly selected and the abundance of arthropods was visually assessed on eight branches of different crown positions of each selected host tree. The arthropods on understory vegetation were assessed on two transects along diagonals of each plot using sweep netting (25 sweeps each, i.e., 50 sweeps in total). As arthropod abundance can change with weather conditions, the surveys were conducted on warm, calm, and sunny days between 10:00 and 12:00.

Ground-dwelling arthropods (e.g., ground beetles and spiders) were surveyed with pitfall trapping. Within each plot, five traps were installed 10 m apart on a transect across plot center. Plastic cups were inserted into ground using a soil auger and the top surface of each cup are flush with soil surface. Each trap was filled with a water solution of 120 ml of 50% ethylene glycol as a temporary preservative and a few drops of colorless, odorless detergent to reduce the surface tension. All pitfall traps were emptied weekly within a month before the artificial flood period. All arthropod samples were preserved in 70% ethanol for species identification at lower taxonomic level to separate arthropods into insect herbivores and predators. The orders Mantodea, Araneae, and Neuroptera, and family Carabidae were predators. Insect herbivores included chewers (i.e., the orders Orthoptera and Lepidoptera, and family Chrysomelidae), leaf miners (i.e., family Lyonetiidae), and leaf-sucking insects (i.e., family Cicadellidae).

### 2.5 Leaf damage and leaf trait measurements

Insect herbivory starts earlier in the spring and their damage would influence MLSD infestation later in the summer (Wang et al., 2021). The five host trees in each plot for arthropod survey were used for assessing cumulative insect damage (Morante-Filho et al., 2016; Castagneyrol et al., 2019; Ruiz-Carbayo et al., 2020) in early June (peak of defoliation) and late August (end of defoliation) of sampling year. Four branches in opposite directions, two from lower crown and two from upper crown, were randomly sampled from each tree using pruning shear/pole pruner. A total of 80 leaf sample were used with 20 leaves randomly sampled from each branch. A transparent plastic sheet with grids of 0.5 × 0.5 cm^2^ was overlaid on sampled leaves to measure leaf damage. The insect damage was calculated as the percent leaf area consumed by insect herbivores. As insect herbivory in early June was similar among plots, only late August data were used. Leaf gallers and rollers were rare; insect damage on host trees only included leaf-chewers, leaf-miners, and leaf-sucking insects.

Disease survey was conducted in late August during the peak of *D. populi* infection shown as small brownish spots (Wang et al., 2022). Four branches were collected from the same five host trees using the same sampling method above. Five leaves were randomly sampled from each branch and the percentage of leaf area infected by fungal pathogens was visually assessed by seven damage classes: 0%, 1-5%, 6-10%, 11-25%, 26-50%, 51-75%, and 76%-100% (Hantsch et al., 2013, 2014; Field et al., 2020). Mean percentage pathogen damage at individual tree level (i.e., 20 leaves) was calculated based on the median of each damage class.

Additional 10 fully expanded, mature, and undamaged leaves per host tree were sampled to determine leaf area and specific leaf area (SLA), common leaf morphology traits that reflect nitrogen leaf content and susceptibility to insects or pathogens (Toome et al., 2010; Ruiz-Carbayo et al., 2020). Fresh leaves totally flat on the worksurface were photographed with a scale and measured for leaf area using ImageJ software. Leaves were then dried for 24 h at 60°C and weighed for dry weight (DW) to calculate SLA from leaf area (cm^2^) and leaf DW (g).

### 2.6 Microclimate conditions

At the center of each plot, a weather station was set up to record relative humidity and temperature at hourly intervals during the study period. A microclimate data logger was installed on a wood pole 2 m above the ground level and covered by a protective solar shield (Keyu Corporation, Jinan, Shandong, China). Two microclimatic variables were determined. Heat exposure was calculated as the cumulative number of hours above 30 °C between June and September. The 30 °C was chosen as insect herbivores and pathogens decrease with increasing exposure to high temperatures (Sabburg et al., 2015; Wojda, 2017) due to direct mortality and more leaf abscission (Mech et al., 2018). Warm and wet conditions were determined as the mean number of hours between 16 °C and 22 °C on wet days throughout the entire study period (Dillon and Meentemeyer, 2019). A wet day was the day when daily average relative humidity was greater than 80%. Therefore, “warm and wet conditions” represent an environment of moist and high temperature that favor insects and pathogens.

### 2.7 Statistical analyses

To select variables with high explanatory power and deal with multicollinearity, we modelled insect herbivory and pathogen infection separately as functions of all measured variables using the least absolute shrinkage and selection operator (Lasso) (function ‘glmmLasso’ in R package *glmmLasso*; Groll and Tutz, 2014; Groll, 2022). The parameter controlling the extent of shrinkage (lambda) was selected by fitting a sequence of models starting from one with a large enough lambda value to shrink all trait estimates to zero and progressing to a small lambda value where all trait coefficients were nonzero. All predictors were scaled and centered before inclusion in the model to ensure the emerging coefficients were comparable (Schielzeth, 2010). The model fitting was done using a Gaussian distribution. Moreover, variance inflation factor (VIF) values of the remaining five predictors were calculated for each leaf trait, and were found to be < 2.0, indicating that multicollinearity between predictors was not a problem.

Separate generalized linear mixed-effect models (GLMMs) were fitted to examine effects of forest loss, tree community attributes, and microclimates on leaf traits and insect and pathogen damage (function ‘glmer’ in R package *lme4*; Bates et al., 2024). The nonzero coefficients variables selected by the Lasso regression were used as fixed effects for insect and pathogen damage (**Table 1**). Plot identity was treated as a random effect to account for the non-independence of measurements from the same plot. Insect herbivory was also included as a fixed effect in the full models of pathogen infection. To simplify models, we used an information-theoretic approach to multi-model inference. For each response variable, a top set of models (ΔAICc < 2) were selected via Akaike’s Information Criterion corrected for small sample sizes (AICc) (Grueber et al., 2011). Averaged parameter estimates were produced from this top set of models using the “model.avg” function in R package *MuMIn* (Bartoń, 2023). This method reduces model selection bias and is appropriate for determining predictors that have the strongest effect (Nakagawa and Freckleton, 2010). Both marginal R^2^ ( ${\text{R}}_{\text{m}}^{2}$ ) for fixed effects and conditional R^2^ ( ${\text{R}}_{\text{c}}^{2}$ ) for both fixed and random effects were calculated. Selected models were checked for spatial autocorrelation based on Moran’s I test for the response variable and the residuals from the fitted models (function ‘moran.test’ in R package *spdep*; Bivand, 2024). There was no spatial correlation in the modeled response variables (P>0.05), and the model residuals showed no significant spatial autocorrelation, indicating that the models accounted for spatial correlation in leaf traits and insect and pathogen damage.

**Table 1:** Predictors with significant and nonzero coefficients in Lasso regression analyses for insect herbivory and pathogen infection.

Predictors	Insect herbivory				Pathogen infection			
	Estimate	SE	*z*	*P*	Estimate	SE	*z*	*P*
(Intercept)	4.65	0.18	25.45	< 0.001	4.49	0.18	24.61	< 0.001
Forest loss	0.62	0.13	7.62	< 0.001	0.04	0.02	0.94	0.034
Warm and wet conditions	0.03	0.16	0.18	0.853	0.37	0.16	5.75	0.016
Heat exposure	0				0.02	0.09	0.27	0.783
Tree diversity	-0.15	0.08	-0.68	< 0.001	-0.5	0.26	-4.96	< 0.001
Host density	-0.18	0.03	-7.09	0.012	-0.15	0.02	-6.14	0.028
Specific leaf area	0.44	0.12	3.23	< 0.001	0.19	0.13	4.03	< 0.001
Leaf area	-0.04	0.01	-4.57	0.025	-0.02	0.01	-2.28	0.224
Herbivore abundance	0.01	0.02	0.04	0.743	0			
Predator abundance	-0.03	0.01	-1.33	0.296	0			

Significant values P < 0.05 are in bold.

**Figure 2:**
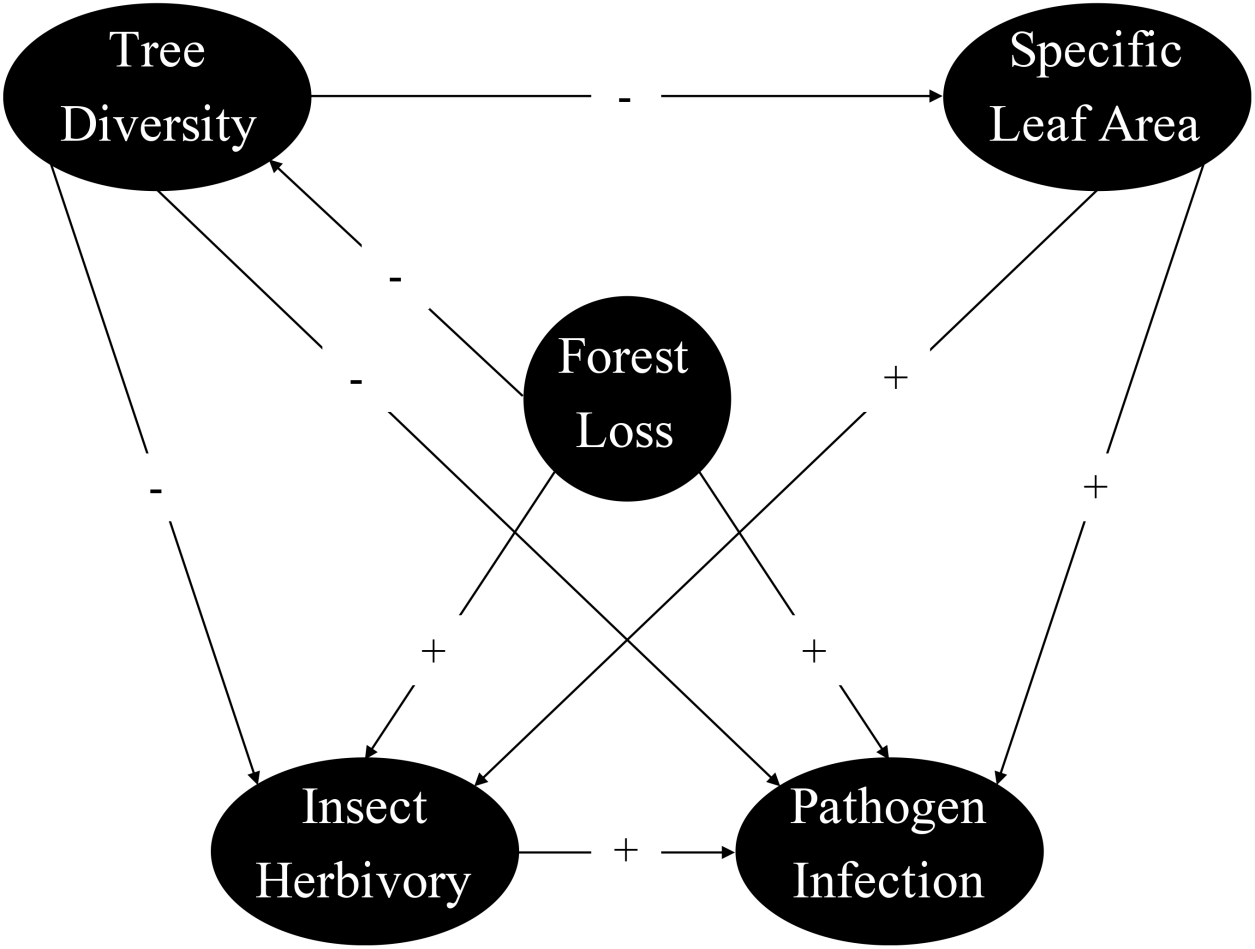
Conceptual model describing the direct and indirect relationships between forest loss, tree diversity, specific leaf area, insect herbivory, and pathogen infection in laurel poplars. Positive and negative hypothetical relationships are illustrated by “+” and “−” on the arrows.

Structural equation modeling (SEM) was used to assess the hypothesized direct and indirect effects of forest loss on insect and pathogen damage that would involve complex relationships (Shipley, 2009; Grace et al., 2010). The *piecewiseSEM* package in R permits the inclusion of hierarchical data by piecing multiple mixed-effects models into one causal framework (Lefcheck, 2016; Lefcheck et al., 2023). We combined component models, accounting for overdispersion where necessary, for direct (e.g., forest loss → insect herbivory; tree diversity → pathogen infection) and indirect relationships (e.g., tree diversity → leaf trait → insect herbivory or pathogen infection) into one causal network (**Figure 2**). For variables repeatedly measured at each plot, we used mixed-effects models with plot as random effects to account for cross-replication and correlation among the measurements from the same plot. We first established a model framework without forest loss-leaf trait relationships. The overall fit of the initial SEM was assessed using Shipley’s test of direct separation to determine the probability of an informative path missing from the hypothesized network (Shipley, 2009). Models were rejected when a χ^2^ test of Fisher’s *C*-statistic fell below the significance level (*P* < 0.05), indicating that the model was inconsistent with the data. Missing forest loss-leaf trait relationships were automatically detected and eventually included in the final models. All statistical analyses were conducted using R software v.4.3.3 (R Development Core Team, 2024).

## 3 Results

Across all 30 plots, 80% of the laurel poplar leaves showed signs of insect herbivory and the average leaf area loss was 10.70% ± 2.25% (mean ± SD). Comparatively, 59% of the laurel poplar leaves showed signs of pathogen infection and the average leaf area infested was 17.68 ± 3.92% (mean ± SD). A total of 2619 predatory arthropods (87.30 ± 37.14 per sample plot) and 1455 herbivorous insects (48.50 ± 21.40 per sample plot) were collected.

Out of the 9 biotic and abiotic variables measured, five were included in the final Lasso regression model of insect damage. Both forest loss and SLA were positively associated with insect damage (**Table 1**), whereas tree diversity, host density, and leaf area were negatively associated with insect damage (**Table 1**). Lasso regression models of pathogen infection also identified five potentially important predictors, including positive effects by forest loss, warm and wet conditions, and SLA, and negative effects by tree diversity and host density (**Table 1**). Both forest microclimates and arthropod abundance did not significantly affect insect herbivory. Heat exposure, leaf area, and arthropod abundance were not significant predictors of pathogen infection.

**Figure 3:**
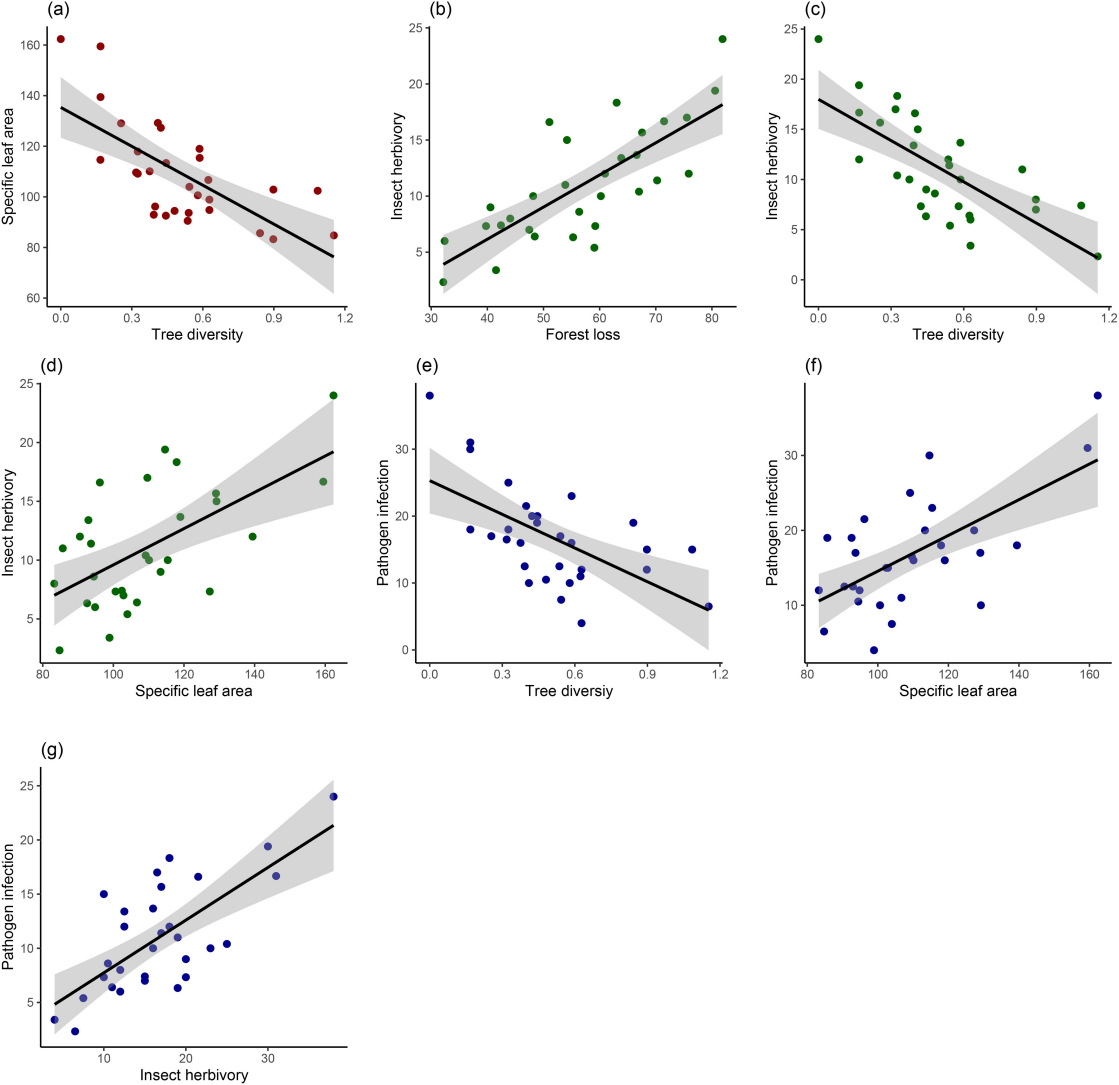
Results of GLMMs testing the effects of **(a)** tree diversity on specific leaf area, **(b)** forest loss on insect herbivory, **(c)** tree diversity on insect herbivory, **(d)** specific leaf area on insect herbivory, **(e)** tree diversity on pathogen infection, **(f)** specific leaf area on pathogen infection, **(g)** insect herbivory on pathogen infection.

For poplar leaf traits, only tree diversity negatively affected SLA (estimate ± SE, -0.22 ± 0.05, P<0.001) (**Table 2**, **Figure 3a**). Insect herbivory decreased with the increase of tree diversity (-0.19 ± 0.11, P=0.002) (**Figure 3c**) and increased with the increases of forest loss (0.70 ± 0.12, P<0.001) (**Figure 3b**) and SLA (0.66 ± 0.09, P=0.022) (**Figure 3d**). Neither of host density (-0.25 ± 0.08, P=0.122) and leaf area (-0.04 ± 0.03, P=0.641) had significant effects on insect herbivory (**Table 2**). Similarly, pathogen infection decreased with tree diversity (-0.30 ± 0.13, P=0.031) (**Figure 3e**) and increased with SLA (0.21 ± 0.16, P=0.004) (**Figure 3f**). Pathogen infection also increased with insect herbivory (0.40 ± 0.13, P=0.004) (**Figure 3g**) but did not vary significantly with forest loss (0.15 ± 0.07, P=0.130), warm and wet conditions (0.45 ± 0.12, P=0.110), and host density (-0.07 ± 0.11, P=0.545) (**Table 2**).

**Table 2:** Results of GLMMs testing the effects of forest loss, microclimates, and tree community attributes on poplar leaf traits, insect herbivory, and pathogen infection.

Response variables	Predictors	Estimate	SE	P	$R_{m}^{2}$ ( $R_{c}^{2}$ )
Leaf area	Intercept	4.75	0.05	0.216	0.37(0.39)
	Forest loss	0.07	0.02	0.916	
	Warm and wet conditions	0.07	0.04	0.321	
	Heat exposure	-0.08	0.04	0.238	
	Tree diversity	0.01	0.02	0.869	
	Host density	-0.09	0.05	0.183	
Specific leaf area	Intercept	4.69	0.02	< 0.001	0.53(0.53)
	Forest loss	0.03	0.07	0.643	
	Warm and wet conditions	0.07	0.05	0.121	
	Heat exposure	-0.04	0.04	0.394	
	Tree diversity	-0.22	0.05	< 0.001	
	Host density	0.08	0.04	0.855	
Insect herbivory	Intercept	2.39	0.09	< 0.001	0.71(0.77)
	Forest loss	0.70	0.12	< 0.001	
	Tree diversity	-0.19	0.11	0.002	
	Host density	-0.25	0.08	0.122	
	Leaf area	-0.04	0.03	0.641	
	Specific leaf area	0.66	0.09	0.022	
Pathogen infection	Intercept	2.79	0.05	< 0.001	0.66(0.67)
	Forest loss	0.15	0.07	0.130	
	Warm and wet conditions	0.45	0.12	0.110	
	Tree diversity	-0.30	0.13	0.031	
	Host density	-0.07	0.11	0.545	
	Specific leaf area	0.21	0.16	0.004	
	Insect herbivory	0.40	0.13	0.004	

Significant values (P < 0.05) are in bold; ${\text{R}}_{\text{m}}^{2}$ = marginal R^2^ with fixed effects only; ${\text{R}}_{\text{c}}^{2}$ = conditional R^2^ with both fixed and random effects.

**Figure 4:**
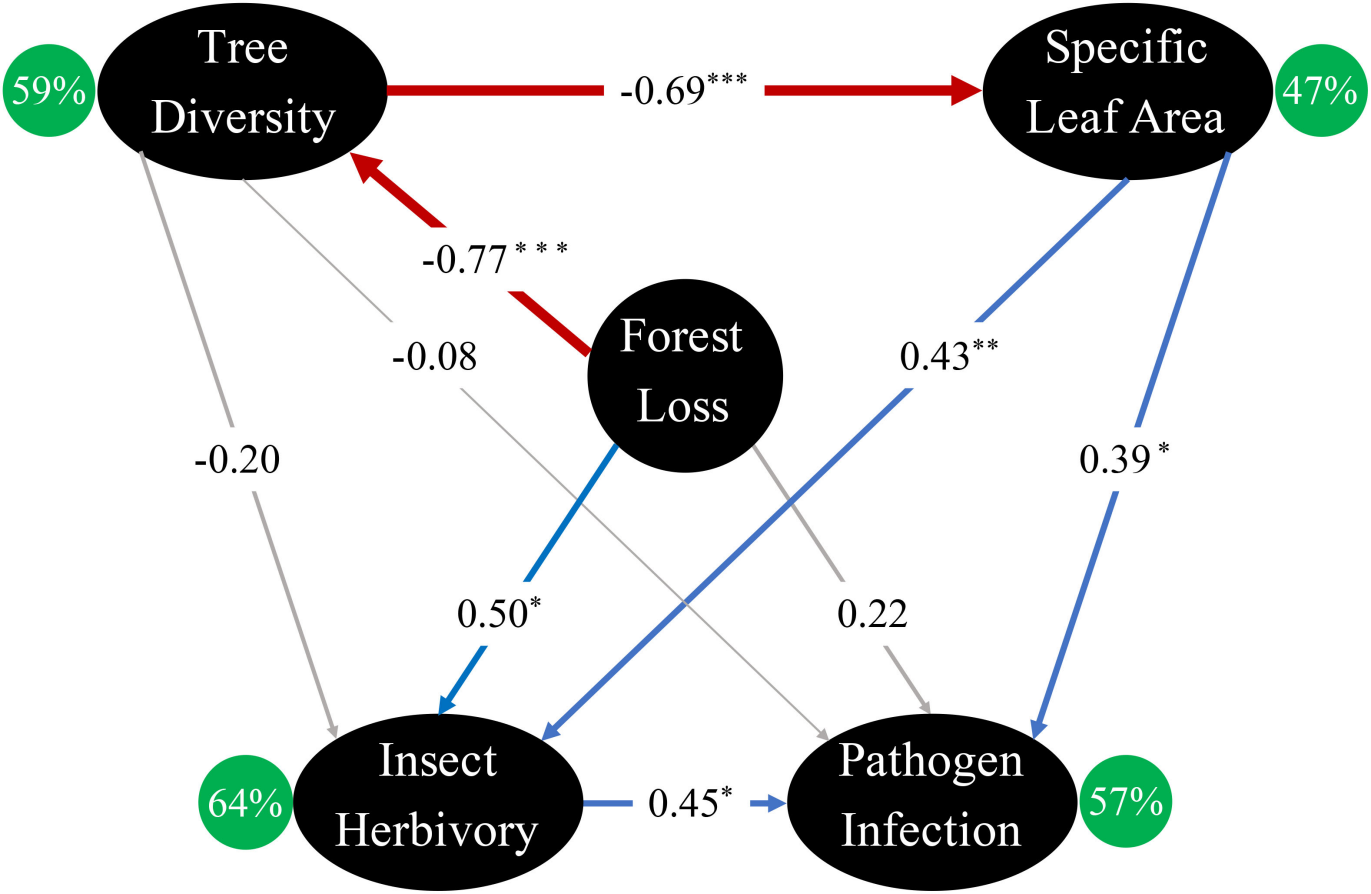
Best-fitted structural equation model illustrating the direct and indirect relationships between forest loss, tree diversity, specific leaf area, insect herbivory, and pathogen infection in laurel poplars. Significant positive and negative relationships between nodes are shown in blue and red, respectively. Insignificant relationships are shown in gray lines. *R* ^2^ of each response variable is shown inside green circle. Standardized path coefficients are shown on the arrows, and the width of the arrow corresponds to the absolute value of each coefficients. Statistical significance^*^.p<0.05; ^**^. p<0.01; ^***^. p<0.001.

Among the 9 pathways in the best model, 6 were significant (**Figure 4**). Forest loss was directly and indirectly associated with insect herbivory, but only indirectly associated with pathogen infection (Chi-square=0.353, *P*=0.553, CFI=0.997, RMSA<0.001). The best-fitted model explained 59% of the variation in tree diversity, 47% of the variation in SLA, 64% of the variation in insect herbivory, and 57% of the variation in pathogen infection (**Figure 4**). Specifically, forest loss directly decreased tree diversity (β=-0.77, p<0.001), which negatively affected SLA (β=-0.69, p <0.001). As a result, insect herbivory was positively associated with forest loss (β=0.50, p=0.037) and SLA (β=0.43, p=0.008) and pathogen infection was positively associated with SLA (β=0.39, p=0.024). Finally, insect damage was directly and positively associated with pathogen infection (β=0.45, p=0.018) (**Figure 4**).

## 4 Discussion

### 4.1 Effects of forest loss on insect herbivory

In line with our hypothesis, plot with higher forest loss was associated with greater damage by insect herbivores in natural forests, consistent with the Resource Dilution Hypothesis (Guimarães et al., 2014; Rossetti et al., 2014; Moreira et al., 2016). This finding is consistent with the results of some previous studies (Peter et al., 2015; Dodonov et al., 2016), but not with those of others where sufficient food sources are available (Ruiz-Guerra et al., 2010; Cavaletto et al., 2019) and insect herbivory did not respond to forest loss (Souza et al., 2013). The species richness and density of understory plants were lower in plots with higher forest loss, which may restrict the availability of potential alternative sources and force insect herbivores to feed more on poplar trees (Peter et al., 2015; Morante-Filho et al., 2016). The greater canopy openness in landscapes of high forest loss is also associated with low abundance and high mortality and damage of shade-tolerant species (Laurance et al., 2006; Santo-Silva et al., 2016; Käber et al., 2021), while promoting the proliferation of pioneer plant species (i.e., poplar trees) (Wang et al., 2022). Pioneer species tend to have limited defenses against insect herbivores, relative to late successional species (i.e., the lack of bottom-up control), and thus suffer a greater insect herbivory damage in fragmented forest landscape (Chen, 2008; Züst and Agrawal, 2017).

Our results suggested forest loss exerted an indirect effect on insect herbivory, via the simplification of forest structure (i.e., lower tree diversity), as suggested by others (Cayuela et al., 2006; Morante-Filho et al., 2016; Rocha-Santos et al., 2016). The low tree diversity was associated with greater SLA, consistent with the observed pattern of associational resistance in mixed species forests (Muiruri et al., 2019; Jactel et al., 2021). In the study area, laurel poplars are the tallest trees, meaning that more diverse plots would have laurel poplars of high exposure to sunlight (Wang et al., 2022) and therefore lower SLA (Muiruri et al., 2019; Ruiz-Carbayo et al., 2020). In contrast, laurel poplars in less diverse plots would have less exposure to sunlight and therefore more shade leaves with a larger SLA. Leaves developed under low light environments are known to be more favorable for the growth and development of insect herbivores (Roberts and Paul, 2006; Sack et al., 2006). The indirect influence of forest loss on insect herbivory through tree diversity-SLA relationship belongs to the bottom-up controls that has been reported in small-scale planted mixed forests (Muiruri et al., 2019; Field et al., 2020), but not in natural forests at landscape scale.

### 4.2 Effects of forest loss on pathogen infection

Forest loss indirectly affected pathogen infection through diversity-SLA relationship, consistent with earlier suggestions (Toome et al., 2010; Wang et al., 2022). Plant leaves with higher SLA and thinner cell walls are known to contain more non-structural carbohydrates, which are easily accessible and metabolized by fungi, therefore promoting disease development (Voegele and Mendgen, 2003; Lee et al., 2005; Berger et al., 2007; Toome et al., 2010). The more diverse plots may also help reduce the encounter rate between susceptible host poplars and dispersed spores (i.e., a dilution effect) (Keesing et al., 2006; Dillon and Meentemeyer, 2019). However, such dilution effects of tree diversity may be reduced by forest loss, resulting in increased pathogen damage in fragmented forests. Guégan et al. (2023) emphasized that forest loss contributes to pathogen emergence and spread. However, they focused on emerging pathogens, rather than native forest pathogens. And the indirect effect of forest loss illustrated the important role of tree diversity in reducing pathogen damage. Moreover, we did not find strong effects of forest microclimates on pathogen damage, inconsistent with earlier suggestions (Clarkson et al., 2014; Velásquez et al., 2018). This is likely due to the conditions of our study area where physical and chemical properties of sandy soils and soil moisture are relatively consistent in the river valley forests of the Irtysh River Basin (Song et al., 2024), as forest microclimate is often regulated by soil properties and moisture (Fornoff et al., 2021).

### 4.3 Interactions between insect herbivory and pathogen damage

The MLSD infection was positively related to insect herbivory, consistent with some studies on forest (Hatcher, 1995; Schuldt et al., 2017). Fungal pathogens generally grow faster in plant tissues previously attacked by herbivorous insects due to cell rupture (Gossner et al., 2021). Many insects also carry fungal spores passively by feeding or walking through infected plant area and provide entry ports for pathogen colonization by removal of physical barriers (Wielkopolan et al., 2021). In this study, the early leaf-chewing damage is the main contributor to insect herbivory, which can weaken poplars and promote subsequent attacks by *D. populi* and MLSD infection. In a tree diversity experiment, however, pathogen infection by powdery mildew in oaks was unrelated to the level of leaf chewing damage measured on the same host trees (Field et al., 2020). It may be that the level of defoliation did not significantly modify oak phenology, for instance, by promoting lammas shoot growth and therefore resource availability for powdery mildew pathogen. Therefore, early insect herbivory may promote the outcome of plant interactions with subsequent attackers (e.g., pathogenic fungi) by inhibiting plant growth (Abdala-Roberts et al., 2019).

### 4.4 Absence of top-down effects on insect herbivory

Contrary to our hypothesis and previous studies, we did not find evidence for a top-down control of predatory and herbivorous arthropods on insect herbivory (Castagneyrol et al., 2017). Tree diversity tends to increase predator abundance and reduce herbivore abundance (Vidal and Murphy, 2018; Stemmelen et al., 2022), as suggested by the “enemies” hypothesis that more diverse communities support more stabilized and efficient predators of herbivores (Letourneau, 1987; Staab and Schuldt, 2020). This positive relationship may result from more habitats and food availability and favorable microclimates (Fornoff et al., 2021; Jactel et al., 2021). In our study, microclimates were not associated with poplar leaf traits or community attributes, possibly due to lack of variations in site conditions among plots. Laurel poplar in study region hosts diverse folivorous insects of both specialist and generalist herbivores, which may restrict associational resistance that have stronger effect on specialists (Jactel et al., 2021). However, the lack of association between predators and insect herbivores does not necessarily implies a lack of top-down control by predators on insect herbivory, due to possible obscuration by strong bottom-up control (Castagneyrol et al., 2017), as found in this study. Further studies are required to elucidate tree diversity-predator-insect herbivore relationship by distinguishing specialist and generalist herbivores in landscapes with high forest loss.

## 5 Conclusions

Our study indicates that landscape-scale forest loss in natural forests directly and indirectly increased insect herbivory but only indirectly increased pathogen infection, and that such associational effects were mediated by the variations in SLA. Pathogen infection was positively associated with insect herbivory. Although no evidence was available to suggest a top-down control of predatory and herbivorous arthropods on insect herbivory through positive tree diversity-predator-insect herbivore relationships, forest loss can weaken bottom-up controls of insect herbivory through diversity- antiherbivore defense mechanisms. Overall, our study illustrates the importance of tree diversity and leaf traits (e.g., SLA) in enhancing resistance to insect and pathogen damage and maintaining health of plantations and natural forests. Accounting for the degree of herbivore specialization in future study on insect and pathogen damage would improve our ability to disentangle the mechanisms underlying associational effects in landscape with higher forest loss.

## Data Availability

The raw data supporting the conclusions of this article will be made available by the authors, without undue reservation.
